# Microsecond X-ray reflectometry

**DOI:** 10.1107/S1600576726004449

**Published:** 2026-06-24

**Authors:** Michael Haberl, Maximilian Eder, Erwin Pfeiler, David Schumi-Mareček, Florian Bertram, Petr Mikulík, Stefan Kowarik

**Affiliations:** a Physikalische Chemie, Graz University, Heinrichstraße 28, Graz, Steiermark 8010, Austria; b Deutsches Elektronen-Synchrotron DESY Notkestraße 85 22607 Hamburg Germany; c Department of Condensed Matter Physics, Faculty of Science, Masaryk University, Kotlářská 2, Brno 61137, Czechia; Montanuniversität Leoben, Austria

**Keywords:** X-ray reflectometry, fast measurements, thin films, Anscombe transform, galvanometer scanning

## Abstract

We accelerate synchrotron X-ray reflectometry (XRR) by more than an order of magnitude and demonstrate the acquisition of full reflectivity curves within 213 µs over a *q*_*z*_ range of 0.05 to 0.35 Å^−1^ at 0.001 Å^−1^ resolution. This is achieved by rapidly sweeping the incidence angle with a high-speed galvanometer and recording the reflected beam on an area detector. The method preserves a monochromatic parallel-beam geometry, requires only straightforward geometric and exposure time corrections, and yields quantitative agreement with standard XRR in thickness, density and roughness. At the shortest acquisition times, the photon statistics enter the low-count Poisson regime, where conventional least-squares fitting becomes biased. We show that applying an Anscombe variance-stabilizing transform restores near-Gaussian error behavior and significantly improves fitting robustness. Our approach benefits not only ultrafast XRR but reflectometry data in general at large *q*_*z*_, where count rates are intrinsically low. This advance enables time-resolved studies of thin-film growth, diffusion, photoswitching and other rapid kinetic processes.

## Abstract 2

X-ray reflectometry is accelerated to 213 µs net exposure time using galvanometer scanning, which enables unprecedented temporal resolution for studying dynamic thin-film processes.

## 1. Introduction

X-ray reflectometry (XRR) is a powerful and widely used technique for determining layer thicknesses, electron densities and interfacial roughness in thin films, multilayers and bulk structures. XRR probes the specular reflection of X-rays and achieves sub-ångström precision even for ultrathin layers, and therefore it is a standard tool in materials science, chemistry and semiconductor research (Daillant & Gibaud, 2009 ▸; Pietsch *et al.*, 2004 ▸; Skoda, 2019 ▸). Conventional XRR employs an angle-dispersive, monochromatic parallel-beam geometry with high-precision scans of the incident angle ω and detector angle 2θ. However, the required small step sizes and long integration times lead to acquisition times of minutes to hours. Although time resolutions as low as 200 ps have been reported using pump–probe schemes (Nüske *et al.*, 2011 ▸), these methods are limited to highly reversible processes and require long total measurement times. With minimal hardware modifications, continuous acquisition schemes such as the FLYSCAN architecture (Medjoubi *et al.*, 2013 ▸) have reduced synchrotron XRR acquisition times to about 10 s per reflectivity curve (Mocuta *et al.*, 2018 ▸). Nevertheless, measurements on the second timescale remain too slow to capture many dynamic and irreversible processes.

To overcome these limitations, a variety of fast XRR approaches have been developed, which can be grouped into two main classes. The first retains the parallel-beam angle-dispersive geometry but increases the speed of mechanical motion. Examples include rotating stages with wedge-shaped sample holders, where rotation about an axis nearly perpendicular to the incident beam continuously changes the effective incidence angle without stepwise repositioning (Lippmann *et al.*, 2016 ▸; Schumi-Mareček *et al.*, 2025 ▸). These methods yield reflectivity curves in the 1 to 100 ms range while preserving the conventional optical geometry and have been applied to studies of structural changes in polymer thin films (Lippmann *et al.*, 2016 ▸) and dynamic thin-film growth such as spin coating (Schumi-Mareček *et al.*, 2024 ▸) and organic mol­ecular beam deposition (Schumi-Mareček *et al.*, 2025 ▸).

The second class avoids mechanical motion entirely by replacing the angular scan. In angle-dispersive fixed-incidence methods, beam-shaping optics generate an angular fan of incident rays that are recorded simultaneously on an area detector. Polycapillary-based implementations (Joress *et al.*, 2018 ▸; Joress *et al.*, 2019 ▸) achieve millisecond-scale XRR by simultaneously sampling a broad range of incidence angles, with full reflectivity curves acquired in 10 to 100 ms. In some cases, curved samples allow simultaneous access to a range of incidence angles and enable collection of reflectivity curves in as little as 2 ms (Stoev & Sakurai, 2013 ▸; Liu *et al.*, 2017 ▸). Alternatively, energy-dispersive XRR employs a white beam and an energy-resolving detector at fixed incidence to probe a *q* range in parallel (Nakano *et al.*, 1978 ▸; Metzger *et al.*, 1994 ▸; Bhattacharya *et al.*, 2003 ▸; Kowarik *et al.*, 2007 ▸). While this approach also eliminates mechanical motion, it is limited by detector count rates to time resolutions of about 60 to 0.1 s (Bhattacharya *et al.*, 2003 ▸). Although fixed-incidence techniques enable real-time monitoring of structural evolution and thin-film growth, they require specialized optics, non-standard beamline configurations, and careful calibration of the angle- or energy-dependent intensity distribution of the incident beam, and they typically access only a fixed *q* range. As a result, achieving sub-millisecond time resolution in a simple and broadly applicable configuration – without added optics or specialized beam conditions – remains a major challenge.

In this paper, we demonstrate that monochromatic parallel-beam XRR can be extended by sweeping the incident angle ω at kilohertz rates using a high-speed galvanometer scanner while recording the reflected beam on an area detector. This approach preserves the standard parallel-beam geometry, requires only straightforward geometric and exposure time corrections, and is compatible with both synchrotron and high-flux laboratory sources. Accessing the hundreds-of-microseconds regime pushes the limits of current third-generation synchrotron flux and single-photon-counting detector capabilities, resulting in very low photon statistics. To enable quantitative fitting of microsecond XRR data, we apply the Anscombe variance-stabilizing transform, which converts Poisson-limited data into an approximately Gaussian form and allows reliable least-squares fitting in quantitative agreement with conventional measurements. Beyond fast XRR, the Anscombe variance-stabilizing transform provides a generally applicable framework for robust quantitative analysis of X-ray and neutron reflectometry data in low-count regimes.

## 2. Methods

### 2.1. Experimental setup

Achieving microsecond-resolved XRR requires sweeping the incident angle at kilohertz rates, which we accomplish using a galvanometer scanner (galvo). A galvo is a rotary actuator combined with an encoder that enables high-speed positioning over a limited angular range. Feedback control allows rapid and highly precise bidirectional scanning in the kilohertz regime and is commonly used in applications such as laser scanning microscopy (Yoo *et al.*, 2016 ▸) and micromachining (Zimmermann *et al.*, 2015 ▸).

**Figure 1:**
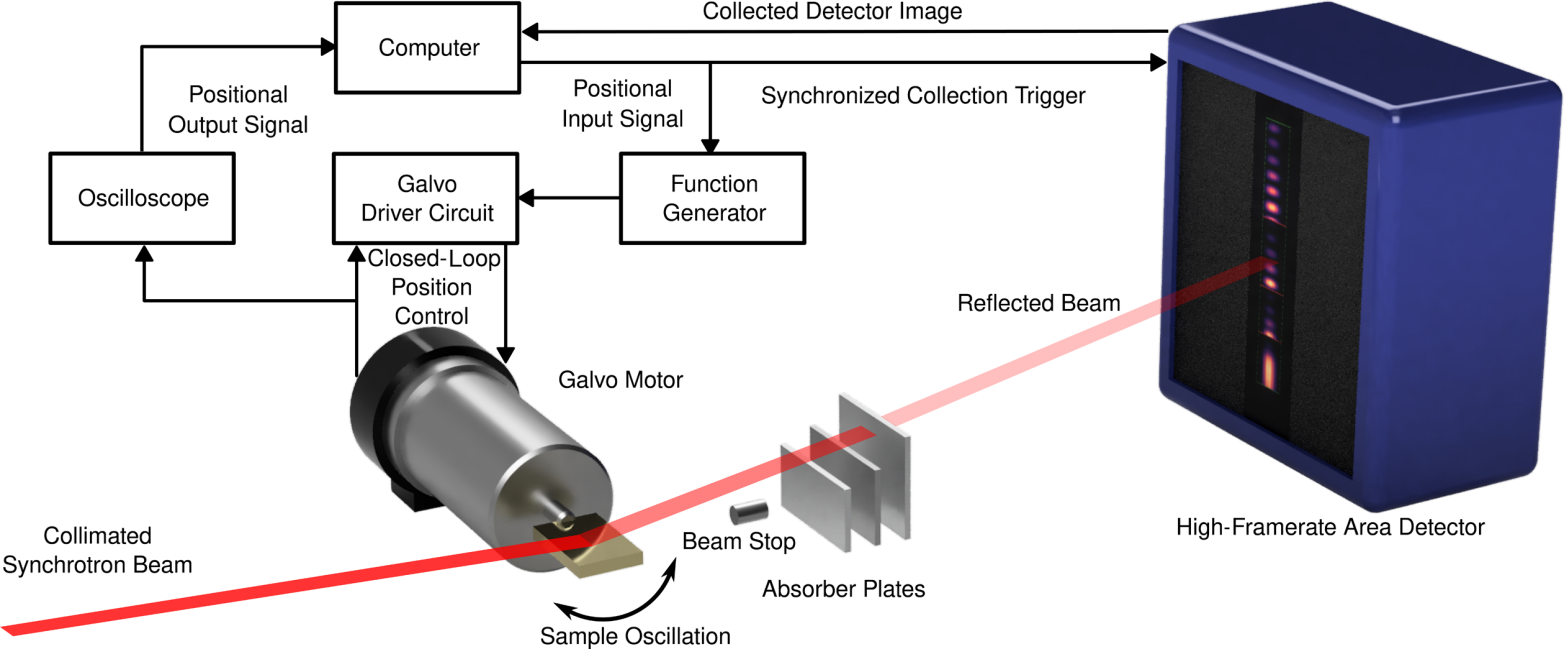
Schematic of the experimental setup for fast XRR measurements. The galvo enables rapid oscillations of the incidence angle ω between incident beam and sample. The absorber plates and detector remain stationary.

Our custom setup for high-speed XRR was implemented at the high-resolution diffraction beamline P08 at PETRA III, DESY (Seeck *et al.*, 2012 ▸). An X-ray energy of 18.00 keV was used, and the beam was collimated to a rectangular cross section of 0.4 × 0.1 mm. As a test sample, we employed a silicon wafer coated with an 80 Å-thick chromium intermediate layer and a 300 Å-thick gold top layer. A schematic of the fast angle-scanning, detection and attenuation setup is shown in Fig. 1 ▸.

The sample stage consists of a DT30Plus (Live Lasersystems GmbH) laser-scanner galvanometer mounted on the beamline hexapod. The sample was glued directly onto the rotational axis of the galvo in place of the original laser mirror, such that the sample surface coincides with the rotation axis. To compensate for the increased mass of the test sample, the feedback control was manually re-tuned using potentiometers on the galvo driver circuit board. A computer sound card (Focusrite Scarlett 2i2) was used as a function generator to supply the analog input signal for the galvo driver, producing triangular or sinusoidal oscillations, as well as detector trigger signals on a second channel. To record the positional feedback signal from the integrated optical encoder, connections on the galvo driver board were tapped, and the analog signal was digitized via the same sound card and stored as a WAV file. Photon collection was triggered twice per galvanometer oscillation period, yielding two complete XRR curves corresponding to the forward and backward scan directions. The detector was configured with a 50 µs readout time per scan, during which no photons were collected.

A stationary Dectris Eiger2 X 1M detector (Donath *et al.*, 2023 ▸) was positioned 157 cm from the galvanometer axis, covering a *q* range from 0 to 0.4 Å^−1^ with a resolution of 0.0010 Å^−1^. Three motorized aluminium absorber plates were arranged in a staggered configuration to attenuate the reflected beam such that the per-pixel count rate remained below approximately 10 photons µs^−1^, thereby avoiding photon pile-up effects in the single-photon-counting detector. A beam stop positioned close to the sample was used to suppress air scattering along the beam path and to protect the detector.

While this setup enables microsecond-resolved XRR, its performance is ultimately constrained by the photon flux and detector capabilities available with current instrumentation. First, the available photon flux at present third-generation synchrotron sources limits the number of photons that can be collected within the sub-microsecond exposure times required for microsecond XRR. For a 1 ms scan divided into approximately 1000 angular sampling points, pixel exposure times are on the order of 10^−6^ s. Therefore, even fluxes of ∼10^12^ photons s^−1^ yield only a few photons per pixel at large *q*, where the reflectivity decays to intensities of ∼10^−6^. Second, even state-of-the-art single-photon-counting detectors are limited by per-pixel count-rate constraints of ∼10^7^ photons s^−1^ (≈ 10 photons µs^−1^), which restricts the accessible dynamic range at low *q*. Although illumination of several adjacent pixels partially mitigates this limitation, per-pixel count-rate limits remain a key consideration for microsecond XRR. In addition, the detector readout times of 50 µs approach our net exposure time of 213 µs. Therefore, the present experiments operate close to the technical limits of current synchrotron and detector technology, although increased flux from future synchrotron upgrades and the use of charge-integrating detectors may enable even faster XRR acquisition in the future.

A series of fast XRR measurements were recorded with sample oscillation frequencies starting from 50 Hz (9.95 ms per scan) up to 1900 Hz (213 µs per scan). Before each fast XRR measurement, a 10 s continuous image exposure integrating over many forward and backward sweeps of the oscillating stage at a given frequency was recorded to confirm correct operation of the galvo. A triangle wave input signal was initially used to achieve a nearly constant angular speed of the sample, which avoids the need for exposure time corrections. However, the output signal of the galvo’s positional sensor displayed increasing distortions at higher frequencies as the galvo fails to follow the abrupt directional changes at the turning points. Scans with a sample oscillation frequency of 500 Hz and above (less than 1 ms per scan) were therefore performed with a sine wave input signal instead, which yielded a closer match of the waveform between input signal and positional output signal.

A reference XRR scan on the test sample of a gold-coated silicon wafer was recorded with a conventional sample and detector scanning setup. The incidence angle ω of the automatically attenuated incident beam on the centered sample was scanned in steps of 0.005° with the beamline goniometer, and the reflected photons were collected for 1 s per step with a Dectris Pilatus 100K area detector centered at the angle 2θ = 2ω of the specular reflection.

### 2.2. Data extraction and normalization

**Figure 2:**
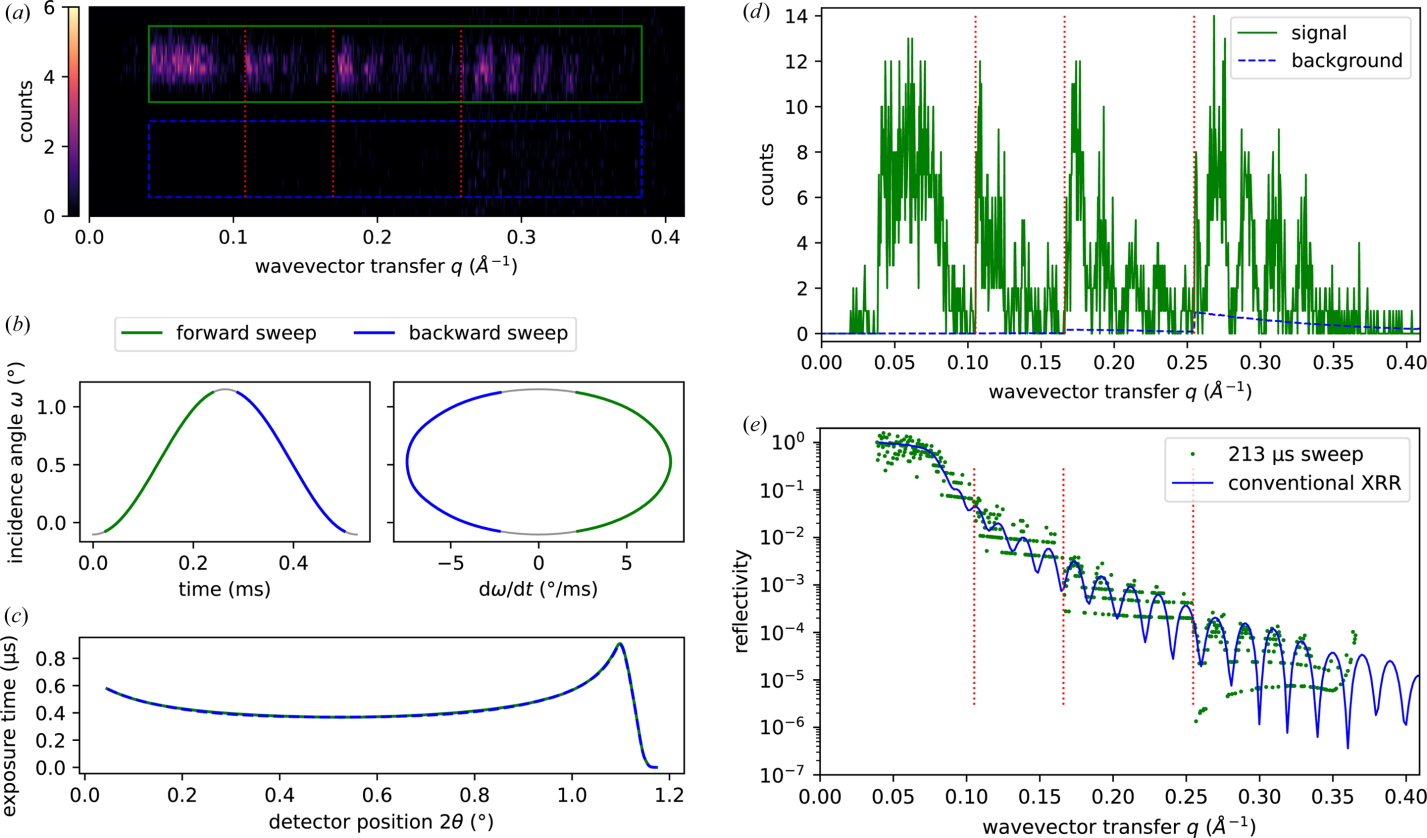
(*a*) Cropped detector image from a 213 µs sweep. Specular reflection and background ROIs are outlined. (*b*) Filtered positional output signal and galvanometer position versus speed. The gray line indicates sample movement during readout. (*c*) Calculated exposure time per pixel row of the detector. The curve for the backward motion is displayed as a broken line for visual clarity. (*d*) Raw counts of the measured reflection signal and averaged background (measured 0.75 mm offset from the reflection plane). The averaged background signal is indicated by a broken blue line. (*e*) Fully normalized fast XRR curve (points) compared with a conventional XRR scan (blue line). The cutoff positions of the stationary absorber plates are indicated as dotted red lines.

We developed a Python-based pipeline to extract the recorded data and apply the necessary normalizations to obtain results consistent with conventional specular XRR. Fig. 2 ▸ illustrates this workflow using a dataset recorded during a 213 µs sweep, including a raw detector image, the corresponding count signal, the averaged background and the fully normalized reflectivity curve. The applied corrections comprise subtraction of an averaged background measured in a second region of interest adjacent to the specular reflection [Fig. 2 ▸(*a*)], attenuation correction for the absorber foils, a geometric footprint correction and normalization for the varying exposure time of individual detector pixel rows arising from the non-uniform angular scan speed of the galvanometer, *e.g.* at the turning points.

The detected intensity *I*_det_(2θ) is modeled as follows from the corrected reflectivity *R*(2θ) as 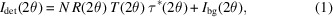
where *N* is a normalization factor, *T*(2θ) accounts for beam attenuation, 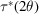 includes exposure time and footprint corrections, and *I*_bg_(2θ) represents the diffuse scattering background.

To determine the exposure time for each detector pixel, the positional output signal of the closed-loop galvanometer, ω(*t*), was filtered using a Fourier-transform-based filter, and its time derivative dω/d*t* was calculated to obtain the angular scan speed [Fig. 2 ▸(*b*)]. This scan speed determines the exposure time of each pixel to the reflected beam [Fig. 2 ▸(*c*)]. Detector deadtime (50 µs per frame) was explicitly accounted for by restricting the exposure window in the reconstruction of the angle-dependent intensity. The effective exposure time depends not only on the instantaneous scan speed but also on the divergence of the reflected beam: ideally, detector pixel rows would be illuminated sequentially during the scan, but beam divergence spreads the intensity over several rows, increasing the effective exposure time and slightly smearing the *q* resolution. Accurate determination of the exposure time is further complicated in our measurements by a slight bending of the sample, which increases the divergence of the reflected beam.

We attribute this increased divergence in the plane of reflection to mechanical and thermal stresses imposed on the sample by its direct attachment to the galvanometer axis. Because the galvanometer was observed to heat up significantly during the measurements, thermal effects are considered the likely cause. Detector scans acquired after the fast XRR measurements (Fig. 5 in the supplementary information) indicate a reflected-beam divergence of approximately 0.15°, which decreases slightly at higher *q*. To account for this effect, the approximate shapes of the rocking curves were extracted from a conventional long-exposure XRR scan and incorporated into the exposure time correction. This unintended sample bending approximately doubled the effective exposure time of individual detector pixels during fast XRR scans, thereby increasing the maximum achievable count rate per scan at the expense of reduced angular resolution. For ideally flat, non-bent samples that are not in direct thermal contact with the galvanometer axis, this additional correction step would not be required. Full details of the exposure time calculation based on the galvanometer positional signal are provided in the supplementary information.

The normalized data were fitted using the differential evolution algorithm implemented in the Python package *refnx* (Nelson & Prescott, 2019 ▸). The gold-coated silicon wafer was modeled as a three-slab system comprising a gold layer, a chromium intermediate layer and a silicon substrate of infinite thickness. The roughness of all three slabs was allowed to vary. The real part of the scattering length density (SLD), the thickness of the gold layer and the thickness of the chromium layer were treated as free parameters. All other SLD values were calculated using the ORSO database (Glavic, 2020 ▸) (ORSO IDs: 14, 24, 79) and kept fixed during fitting.

To estimate parameter uncertainties, Markov chain Monte Carlo (MCMC) sampling was performed with the package *emcee* (Foreman-Mackey *et al.*, 2013 ▸) starting from the initial differential evolution fits in *refnx*. The standard deviation of the measured intensities was approximated as the square root of the photon count and set to unity for zero-count data points, following standard practice (Lass *et al.*, 2021 ▸). Because the reflected beam sweeps continuously across the detector in our measurement scheme, the *q* resolution is assumed to be limited by the width of the reflected beam and the pixel size in the reflection plane, resulting in a resolution of approximately 5 × 10^−4^ to 10^−3^ Å^−1^. Contributions from the intrinsic beam divergence (2 µrad) and the relative wavelength uncertainty (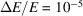) of beamline P08 in collimation mode (Seeck *et al.*, 2012 ▸) are small in comparison and were therefore neglected.

### 2.3. Fitting low-count data using the Anscombe transform

Least-squares fitting of reflectivity data commonly assumes Gaussian-distributed measurement errors. Under this assumption, maximizing the log-likelihood is equivalent to minimizing the weighted sum of squared residuals, where the weights are determined by the estimated variance of the measured intensities (Nelson & Prescott, 2019 ▸). Such weighting is required to account for the heteroscedastic noise inherent to photon-counting data. At very low count rates, however, the Gaussian approximation is no longer valid and the Poisson nature of photon-counting statistics must be treated explicitly (Kirkpatrick & Young, 2009 ▸; Mendenhall, 2018 ▸). Lass *et al.* (2021 ▸) derived Poisson log-likelihood expressions for reflectometry fitting that significantly reduce bias in this regime, but these likelihood formulations are not implemented by default in commonly used reflectivity analysis packages and generally require custom modifications to the optimization routine (Kienzle *et al.*, 2017 ▸; Nelson & Prescott, 2019 ▸; Glavic & Björck, 2022 ▸).

As an alternative, we apply a variance-stabilizing transformation to both the measured XRR data and the corresponding model prior to fitting. In contrast to standard reflectometry workflows (McCluskey *et al.*, 2023 ▸), the measured intensities are not normalized; instead, the model reflectivity is scaled by the relevant intensity correction factors and combined with the (ideally noise-free) diffuse scattering background. After the Anscombe transform has been applied, Poisson-distributed noise is converted into approximately additive Gaussian noise with nearly constant variance (Anscombe, 1948 ▸; Murtagh *et al.*, 1995 ▸), enabling unweighted least-squares fitting and substantially reducing systematic bias in the fitted reflectivity curve (Fig. 7, supplementary information).

Although the use of variance-stabilizing transformations has been reported primarily in imaging applications (Murtagh *et al.*, 1995 ▸; Mascarenhas *et al.*, 1999 ▸; Mäkitalo & Foi, 2013 ▸; Zhang *et al.*, 2017 ▸), it has also been applied in neutron reflectometry for statistical comparison of experimental and simulated data (Durant *et al.*, 2021 ▸). To our knowledge, this approach has not previously been reported for X-ray reflectometry. In this work, only Poisson noise is considered for the transformed data; however, additional uncertainty contributions can be incorporated straightforwardly via error propagation or by using the generalized Anscombe transform (Murtagh *et al.*, 1995 ▸), providing a flexible framework without requiring modifications to the fitting algorithm.

While the Poisson distribution is strongly asymmetric at low count rates and is poorly approximated by either a Gaussian or a log-normal distribution, a Gaussian with unit variance on the Anscombe-transformed scale provides a much closer approximation to the discrete Poisson probability density (Fig. 8 in the supplementary information). The Anscombe transformation stabilizes the variance effectively for expected count rates as low as approximately three; below this threshold, both the mean and the variance begin to deviate slightly from their asymptotic values (Anscombe, 1948 ▸; Freeman & Tukey, 1950 ▸).

## 3. Results

**Figure 3:**
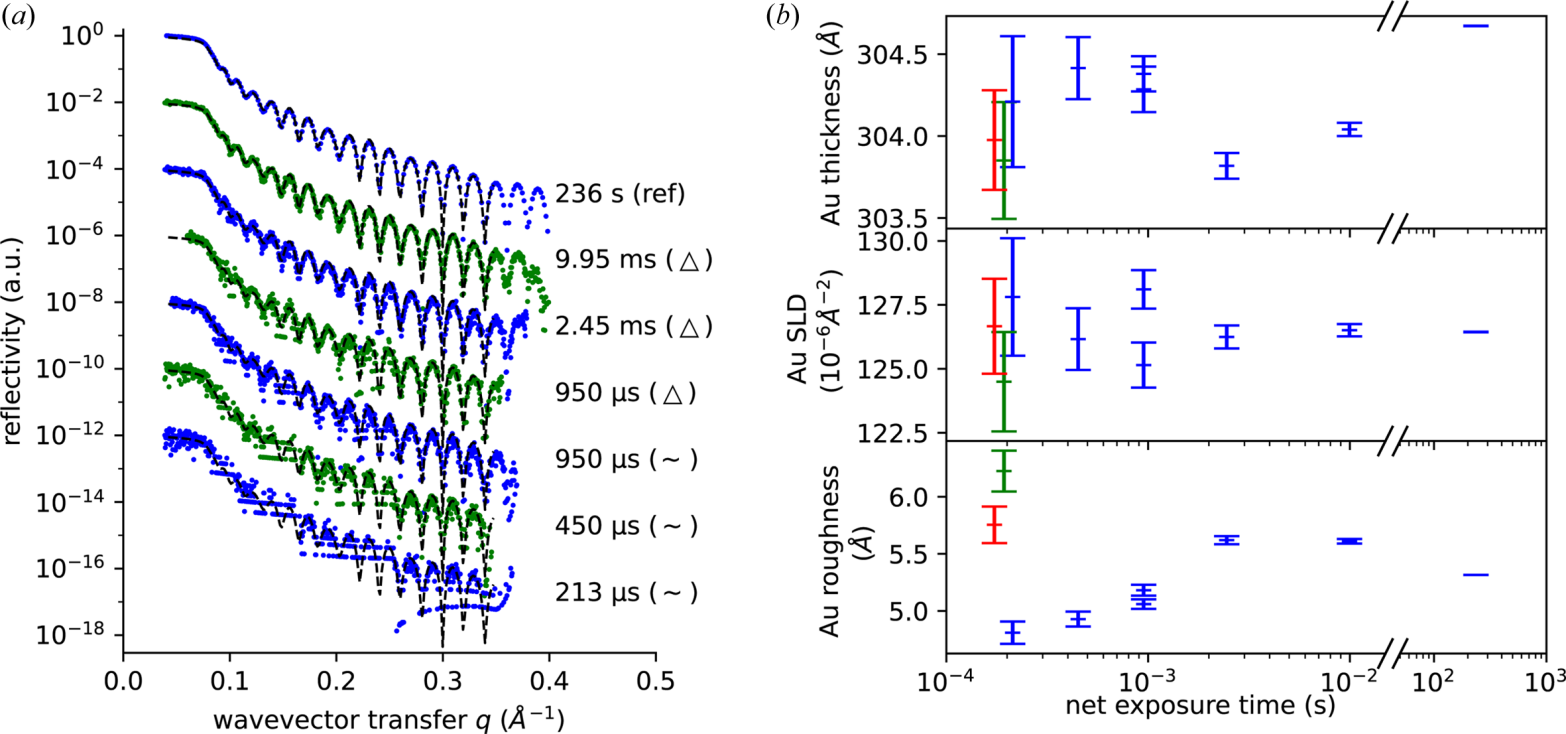
(*a*) Normalized XRR curves with different collection times. ▵ denotes a triangle ω scan and ∼ a sine ω scan. Curves are shifted by 10^2^ with respect to each other for visual clarity. The *refnx* fit of the reference XRR scan (solid line) is overlaid for reference. Constant exposure time was assumed for reference and 9.95 ms fast XRR scans. (*b*) Fitted parameters of the top gold layer for these scans. The median and estimated standard deviation from the MCMC chain are plotted. All scans are fitted after 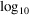 transformation. The fastest scan at 213 µs scan time was additionally fitted untransformed (shown in green) as well as after Anscombe transformation (red); these are plotted with a shift to the left on the *x* axis for clarity.

A selection of normalized reflectivity curves from the same sample measured with different exposure times is shown in Fig. 3 ▸. The fast-scan curves agree well with the conventional XRR reference and its fit (dashed line); however, shorter acquisition times lead to a noticeable increase in noise due to low-count photon statistics. In the fastest scans, the limited dynamic range at low *q* and the lack of photons at high *q* render the Kiessig oscillation minima poorly. Counts become strongly quantized to values of 0, 1 or 2 photons, as evident from the discrete intensity levels after count-time normalization, and even the maxima contain only a few counts. As a consequence, the minima do not visually reach the deep values expected for well-resolved oscillations. Zero-count data points are not displayed on the logarithmic scale.

All datasets shown in Fig. 3 ▸ have been fitted in *refnx* with an identical structure model, and the resulting fit was sampled with the MCMC algorithm. For all scans, the normalized intensities and their estimated standard errors were transformed using 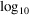; data points yielding non-physical negative reflectivity values after background subtraction were discarded. For the fastest scan series, the additional correction of using the Anscombe transformation was required, as discussed above. The fitted gold layer thickness values shown in Fig. 3 ▸(*b*) demonstrate remarkable consistency between the slow reference scan and the fast galvo-based XRR measurements, with deviations below 1 Å (≈ 0.3%). As expected from increased photon shot noise at shorter exposure times, the uncertainties of the fitted parameters increase with decreasing acquisition time. Nevertheless, both the thickness and the SLD of the top layer remain consistent with values obtained from conventional specular XRR, confirming the quantitative reliability of XRR measurements in the microsecond regime.

**Figure 4:**
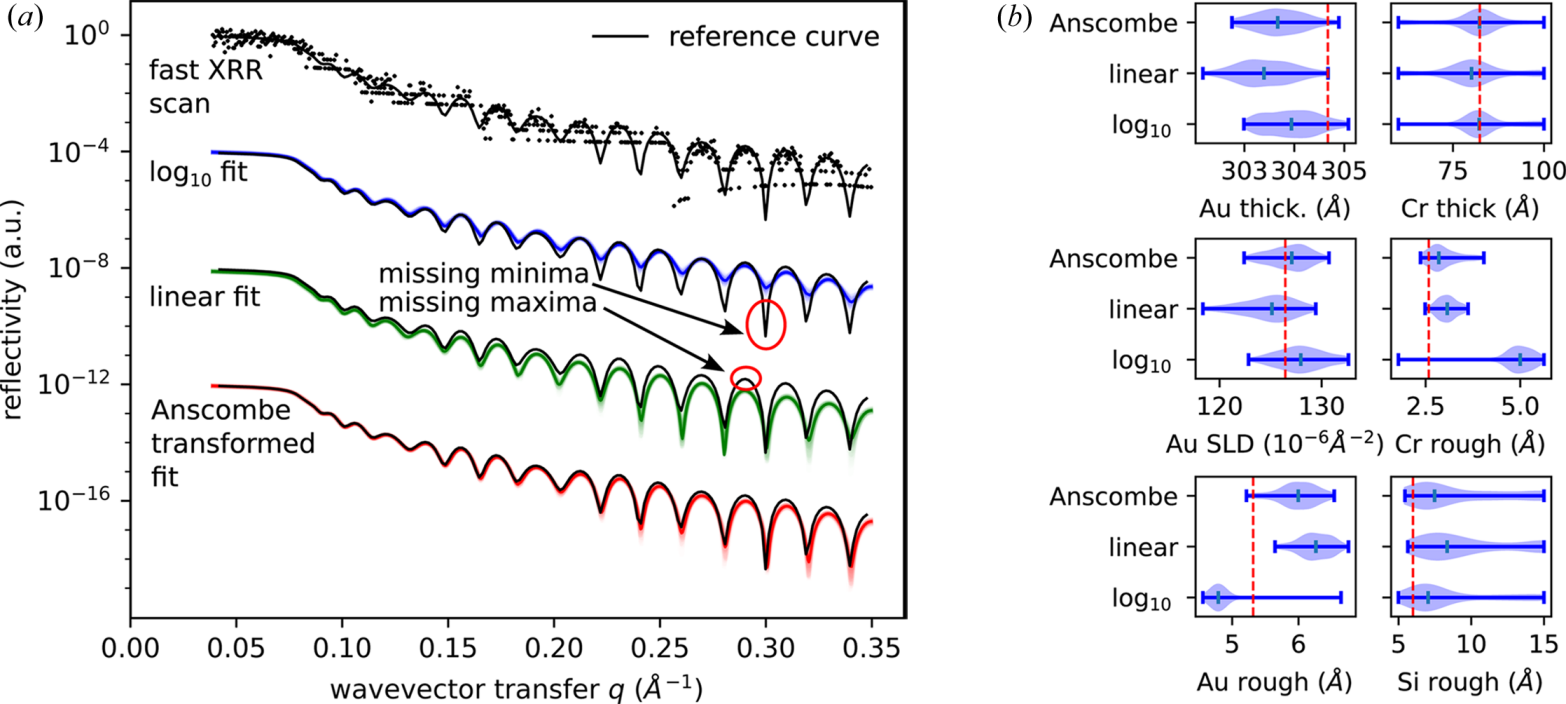
(*a*) Comparison of *refnx* fits of a normalized fast XRR reflectivity curve recorded within 213 µs with different data transformations. Curves are shifted by 10^4^ relative to each other for visual clarity. The range of solutions generated from MCMC samples are displayed as colored shading. (*b*) Distribution of fitted parameters from 100 individual scans with 213 µs exposure time. Fits were performed using identical fitting parameter boundaries in each case with the differential evolution optimizer without MCMC sampling. The reference value from fitting a conventional XRR curve is displayed as the dashed red line.

Fig. 4 ▸ presents representative fits for the fastest scan series with a net exposure time of 213 µs. Although the extracted parameters show reasonable agreement with the reference values, the figure highlights a key limitation: fitting either 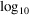-transformed data or untransformed (linear) data leads to systematically different behavior. Specifically, 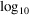-transformed reflectivity fails to reproduce the Kiessig minima accurately, while fitting on a linear scale tends to underestimate the maxima. As a result, neither approach reliably captures both extrema of the reflectivity curve for data with such low photon statistics.

This discrepancy arises primarily from correlations between the estimated variance and the measured intensity introduced by the respective data transformations. An illustrative example of the resulting systematic bias in least-squares fitting of Poisson-distributed count data is shown in Fig. 7 of the supplementary information. For untransformed count data, the bias approaches −1 count, consistent with previous reports (Kirkpatrick & Young, 2009 ▸; Mendenhall, 2018 ▸; Lass *et al.*, 2021 ▸) and with the fitting behavior observed in Fig. 4 ▸. In contrast, weighted least-squares fits of 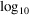-transformed data exhibit a positive bias of approximately +0.5 counts, increasing to nearly +1 count when zero-count data points – which cannot be transformed – are excluded.

In contrast, the 213 µs dataset is fitted substantially better when applying the Anscombe variance-stabilizing transformation (Anscombe, 1948 ▸) to both the raw count data and the model function. Prior to transformation, the model was rescaled to account for attenuation, footprint, exposure time and background contributions according to equation (1). Both the scaling and the transformation were implemented using a custom Transform class in *refnx*. For the Anscombe-transformed fits, the model function *I*_fit_(*q*) was additionally corrected for the asymptotic transformation bias by subtracting 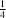 prior to transformation, which results in the least-squares optimization objective given in equation (2):
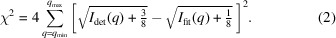
The resulting fits are shown in Fig. 4 ▸(*a*). The Anscombe-transformed fit shows the closest agreement with the reference reflectivity up to *q* = 0.25 Å^−1^, whereas the 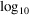-transformed fit deviates in the Kiessig minima and the linear-intensity fit underestimates the maxima. A comparison of fitted parameters obtained from 100 consecutive scans with a 213 µs acquisition time [Fig. 4 ▸(*b*)] indicates that all three approaches yield consistent values for the gold layer thickness (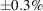) and SLD (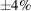). In contrast, the roughness parameters obtained from 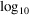-transformed data are systematically shifted, a trend that is also observed for the slower 950 µs and 9.95 ms scans (Table 1, supporting information). Because surface and interface roughness predominantly affect the reflectivity at high *q* (Baumbach & Mikulík, 2009 ▸; Karlsson *et al.*, 2024 ▸), the reduced photon statistics in this region lead to larger uncertainties in the extracted roughness parameters.

The MCMC posterior distributions (see Fig. 9 in the supporting information) further support this interpretation: under the 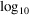 transformation, the Si-substrate roughness exhibits a skewed, non-Gaussian uncertainty distribution, whereas the Anscombe-transformed fit yields a more symmetric and better-constrained posterior. These observations are consistent with the fact that a substantial fraction of the measured intensities in fast scans lie in the Poisson regime, where variance-stabilizing transformations or explicit Poisson-likelihood approaches are essential for obtaining reliable fit parameters.

## 4. Discussion

The fast XRR approach presented here extends conventional monochromatic parallel-beam reflectometry into the sub-millisecond regime while preserving a simple and broadly applicable experimental geometry. In contrast to rotating-stage implementations (Lippmann *et al.*, 2016 ▸; Schumi-Mareček *et al.*, 2024 ▸; Schumi-Mareček *et al.*, 2025 ▸), the galvanometer-based scanning scheme maintains a constant sample azimuth angle throughout the measurement, simplifying alignment and facilitating measurements on standard square or rectangular samples. The accessible angular range can be adjusted electronically via the galvanometer drive amplitude, eliminating mechanical modifications such as exchanging fixed-angle wedges. Combined with stationary beamline optics and a fixed detector position, this makes the method straightforward to implement at existing synchrotron beamlines without specialized hardware.

Because the incidence angle is swept continuously while the detector remains fixed, the measured fast XRR curves correspond to integrated rocking curves rather than purely specular reflectivity at a single detector angle. As shown in earlier work, such integrated curves generally reproduce specular reflectivity oscillations well, although localized intensity deviations may occur in the measured curve (Mocuta *et al.*, 2018 ▸). In the present study, the close agreement between fast and conventional XRR data demonstrates that these effects do not compromise the quantitative determination of thickness, density or roughness within the investigated *q* range, even at the shortest acquisition times.

Reaching acquisition times of a few hundred microseconds inevitably exposes limitations imposed by detector count-rate capabilities and photon statistics. The rapidly scanning reflected beam illuminates individual detector pixels only for a small fraction of the scan, resulting in effective exposure times down to a few tenths of a microsecond. To avoid photon pile-up in single-photon-counting detectors such as the Eiger2 (Dectris), the per-pixel count rate must remain below the specified maximum, which severely limits the dynamic range at low *q* and necessitates strong attenuation. At higher *q*, attenuation can be reduced, but the *q*^−4^ decay of the reflectivity ultimately renders the signal flux limited. These effects manifest as increased statistical uncertainty at high *q* and broader posterior distributions in the MCMC analysis, particularly for roughness-sensitive parameters.

Several routes exist to further improve the performance of fast XRR within these constraints. Optimized attenuation schemes using a larger number of absorber plates, or deliberate broadening of the reflected beam perpendicular to the scattering plane, could distribute photons across more detector pixels and relax per-pixel rate limits. More fundamentally, the technique would strongly benefit from charge-integrating detector technologies such as AGIPD (Henrich *et al.*, 2011 ▸; Allahgholi *et al.*, 2016 ▸), MM-PAD (Gruner, 2017 ▸) or JUNGFRAU (Mozzanica *et al.*, 2018 ▸), which are designed to handle intense, short photon bursts and offer orders-of-magnitude higher dynamic range than single-photon-counting detectors.

Despite these limitations, the high scan rates achieved here enable the acquisition of thousands of full reflectivity curves per second and open new opportunities for studying rapid and irreversible processes. In combination with co-refinement strategies or machine-learning-assisted analysis using parametrized time-dependent models (Mareček *et al.*, 2022 ▸; Schumi-Mareček *et al.*, 2025 ▸), fast XRR provides a powerful route toward quantitative, time-resolved reflectometry on previously inaccessible timescales.

## 5. Conclusion and outlook

We have demonstrated that X-ray reflectometry can be accelerated by more than an order of magnitude into the hundreds-of-microseconds regime using a closed-loop feedback-controlled galvanometer sample stage, while preserving a conventional monochromatic parallel-beam geometry. This approach enables the acquisition of full XRR curves on sub-millisecond timescales using standard beamline optics and straightforward corrections analogous to conventional footprint, absorber and integration-time normalizations. No additional characterization of the incident beam angular or energy distribution is required, nor are specialized wide-band beamlines or dispersive detector concepts necessary. As a result, the method is readily compatible with existing synchrotron beamlines and provides access to time-resolved studies of rapid and irreversible surface and thin-film processes, including interfacial reactions, diffusion, photo-induced structural changes and fast external perturbations. While demonstrated on a static sample as a proof of concept in this work, we plan to apply this method in future studies to fast processes or radiation-sensitive materials, where short exposure times are advantageous, such as high-rate vacuum deposi­tion, photostriction or laser-induced metal interdiffusion. The galvanometer-based setup can be used in vacuum environments with commercially available vacuum-compatible galvos, while applicability to complex sample environments such as liquids remains to be investigated. Similar rapid angular oscillation schemes may also be beneficial for other grazing-incidence scattering techniques to ensure robust sampling of angle-dependent features.

At these extreme acquisition speeds, photon statistics inevitably enter the low-count Poisson regime, making careful statistical treatment essential to avoid systematic fitting biases. We show that applying the Anscombe variance-stabilizing transformation enables reliable least-squares fitting by restoring approximately Gaussian error behavior, yielding results comparable to Poisson log-likelihood approaches while remaining straightforward to implement within existing reflectometry workflows. Importantly, the benefits of this approach extend beyond fast XRR: variance-stabilizing transformations are equally relevant for conventional XRR and neutron reflectometry, particularly at large momentum transfer where count rates are intrinsically low. Together, fast galvanometer-based XRR and statistically robust data treatment provide a broadly applicable framework for quantitative, time-resolved reflectometry across techniques and timescales.

## 6. Related literature

The following references are cited only in the supporting information: Blanton *et al.* (2011 ▸); Toby & Von Dreele (2013 ▸).

## Supplementary Material

Supporting information file. DOI: 10.1107/S1600576726004449/xx5100sup1.pdf

## Data Availability

All data used in this work are available for download on Zenodo (https://doi.org/10.5281/zenodo.18548583).
