# Supplementary material for: Microsecond X-ray reflectometry
Source: J Appl Crystallogr. 2026 Jun 24;59(Pt 4):1112–20. doi: 10.1107/S1600576726004449 (PMC13440043; doi:10.1107/S1600576726004449)
Supplement: Supplementary file 1 [file j-59-01112-sup1.pdf]

JOURNAL OF
APPLIED
CRYSTALLOGRAPHY

Volume 59 (2026)

Supporting information for article:

Microsecond X-ray reflectometry

Michael Haberl, Maximilian Eder, Erwin Pfeiler, David Schumi-Mareček, Florian Bertram, Petr Mikulík and Stefan Kowarik

6 Supplementary Information

6.1 Data Reduction

The 10 s exposure scans with the galvo stage were used to estimate the maximum incidence angle of the sample during a fast XRR series for calibration of the positional signal. The raw positional output signal was filtered using the real-valued fast Fourier Transform (`rfft`) as implemented in **scipy**. A Blackman window was applied to the noisy signal prior to transformation. Frequencies with a magnitude 70 dB lower than the main frequency ($< 10^{-3.5}$) were discarded. A single period of the filtered signal was resampled by a factor of 10^4 and used to calculate the exposure of different pixels.

If the height of the reflected beam is comparable to the vertical pixel size of the detector, the normalization factor of the exposure time for a pixel centered at 2θ can be reasonably approximated as the angular velocity $\frac{\mathrm{d}\omega}{\mathrm{d}t}$ of the of the sample at the angle $\omega = 2\theta/2$ divided by the angular size of the pixel $\Delta 2\theta$. However, with the sample used in this study a significant divergence of the reflected beam of over 0.1° in 2θ was observed (Figure 5), which needs to be accounted for to calculate the effective exposure time of the reflected beam on each pixel from the positional feedback signal of the galvo.

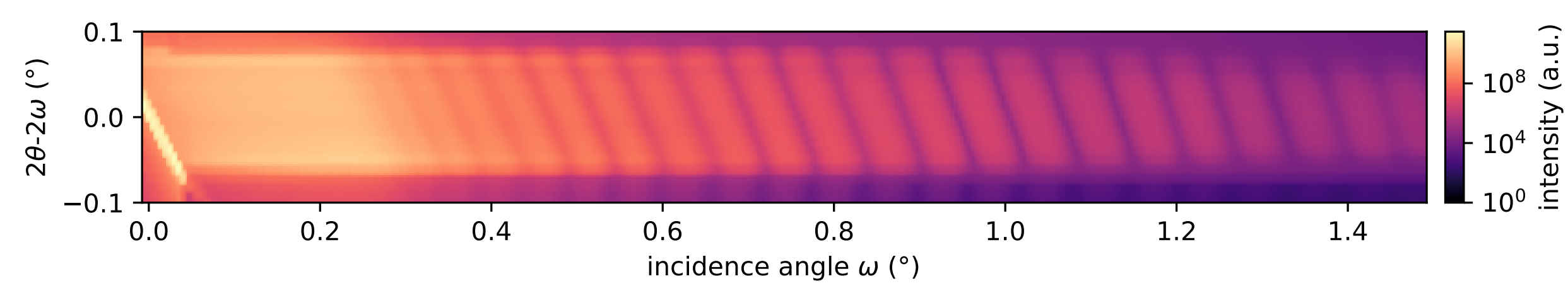

Figure 5: Detector scans of the sample collected after the fast XRR measurement series, attenuation and footprint corrected.

As discussed by (Mocuta *et al.*, 2018), scanning the incidence angle ω and detecting with an area detector corresponds to recording a series of two-dimensional cuts in the reciprocal space. If a stationary area detector sums all images from such a scan, this equals to an integration over ω,

or summing all intensities of the rocking curves. In the summed detector image, the components q_y (normal to the scattering plane) and $\sqrt{q_x^2 + q_z^2} \approx q_z$ (within the scattering plane) remain. The XRR curve is observed at $q_y \approx 0$.

From the recorded feedback signal of the galvanometer scanner, we found that scanning ω with a triangular input signal at higher scan speeds results in a sample motion with rate $\frac{\mathrm{d}\omega}{\mathrm{d}t}$ that deviates increasingly from an expected constant value. This deviation between input and positional output signal shape was reduced with a sine input signal. However, this non-constant rate causes the exposure time of the reflected beam on the detector to be dependent on the incidence angle ω and thus the angular position of the reflected beam 2θ.

The exposure time $\tau(2\theta)$ at a position 2θ on the detector is obtained by integrating the inverse of the galvo angular speed $v(\omega) = \frac{\mathrm{d}\omega}{\mathrm{d}t}$ with the rocking curve of the specular reflection $r(\omega, 2\theta)$ over the incident angle ω in the interval $[\omega_{min}, \omega_{max}]$ during a single detector frame:

$$\tau(2\theta) = \int_{\omega_{min}}^{\omega_{max}} \frac{r(\omega, 2\theta)}{v(\omega)} \, \mathrm{d}\omega \tag{3}$$

The rocking curve intensities $r(\omega, 2\theta)$ are normalized to integrate to unity over ω:

$$\int_{\omega_{min}}^{\omega_{max}} r(\omega, 2\theta) \, \mathrm{d}\omega = 1 \text{ for all } 2\theta \tag{4}$$

Strictly speaking, the beam footprint $a(\omega)$ on the sample is dependent on the incidence angle ω, so this correction is included in the integration over ω to obtain the correction factor $\tau^*(2\theta)$:

$$\tau^*(2\theta) = \int_{\omega_{min}}^{\omega_{max}} \frac{a(\omega)}{b} \cdot \frac{r(\omega, 2\theta)}{v(\omega)} \, \mathrm{d}\omega \tag{5}$$

The footprint $a(\omega)$ is approximated as the length covered by a rectangular beam with height b and an incident angle ω centered on a flat rectangular sample of length d:

$$a(\omega) = \begin{cases} 0 & \text{for } \omega \leq 0 \\ d \cdot \sin\omega & \text{for } 0 < d \cdot \sin\omega < b \\ b & \text{for } d \cdot \sin\omega \geq b \end{cases} \tag{6}$$

These integrals were approximated numerically as sums with step widths equal to the angular size of the detector pixels to obtain the exposure time $\tau(2\theta)$ and correction factor $\tau^*(2\theta)$ for each pixel row on the detector. Resulting values were multiplied by the angular size of the detector pixels to obtain time values in seconds. Pixel positions on the Eiger2 1M detector were calibrated from a diffraction image of silver behenate (CAS RN: 2489-05-6). In the data reduction, functionality of the software package GSAS-II (Toby & Von Dreele, 2013) was used with lattice parameters from

literature (Blanton *et al.*, 2011).

A whole range of rocking curves at various 2θ values can be obtained simultaneously during a single conventional (step-wise, ω-2θ) XRR scan when using an area detector. A Dectris Pilatus 100k was used to capture both the specular reflection and diffuse scattering with slits at the detector side opened slightly wider than the full reflected beam. The position of the primary beam on the area detector is used for a crude calibration of the mobile area detector, mapping pixel position to a 2θ value with a linear function. The detector images were normalized with respect to incident beam intensity and footprint on the sample. Background intensity was subtracted from the normalized intensity. After scaling by q^6, to obtain an approximately constant oscillation amplitude at both low and high q, the Kiessig oscillations were smeared out by applying a rolling average twice over a range of 0.12° in 2θ (covers approximately one Kiessig oscillation). Values at specific points $r(\omega, 2\theta)$ were obtained from this smoothed image by linear interpolation. The reconstructed rocking curves are shown in Figure 6. A visible change in the shape of the rocking curves at higher q can be seen.

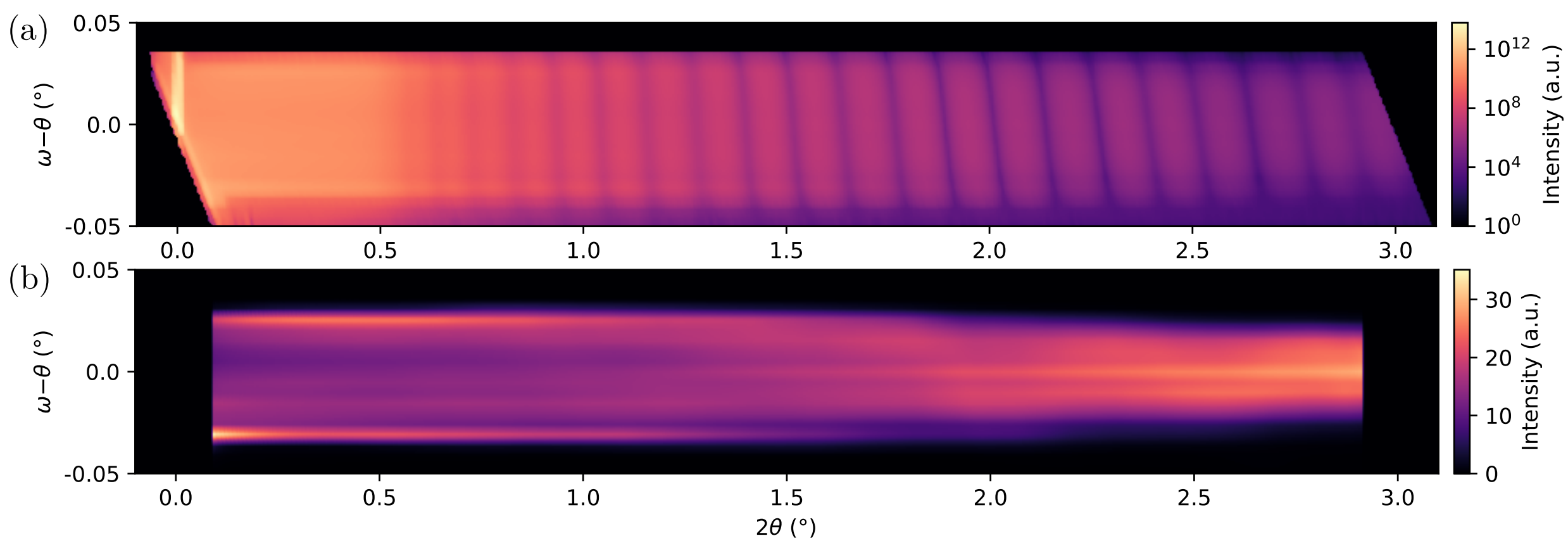

Figure 6: Reconstructed rocking curves of the sample collected after the fast XRR measurement series. (a) Attenuation-, footprint-corrected and diffuse scattering background-subtracted reference scan on a logarithmic scale, Kiessig fringes are well visible. Minima at higher 2θ are not parallel to the primary beam at $2\theta = 0°$. (b) Normalized rocking scans with smeared-out Kiessig Fringes.

6.2 Fitting with Anscombe Transform and Poisson Log-Likelihood

For more careful consideration of count statistics, it is necessary to fit integer photon counts directly (Lass *et al.*, 2021). In this work, this was readily achieved by simply applying all data normalization steps on the fitting model reflectivity function in reverse. The model reflectivity function $R_{\mathrm{fit}}(q)$ was plugged into equation (1) to obtain the rescaled model function $I_{\mathrm{fit}}(q)$. This rescaling is required for fitting with both the Anscombe Transform and Poisson Log-Likelihood and realized using a custom `Transform` class in **refnx**, with all required factors and scattering background intensity provided from additional columns of the dataset file. In the same `Transform` class, the Anscombe Transform is additionally applied to both data and model (with bias correction).

The weighted least-squares optimization goal for data with mean $y(q)$ and standard error $\sigma(q)$ with a model function $\mu(q)$ is defined as (Nelson & Prescott, 2019; McCluskey *et al.*, 2023):

$$\chi^2 = \sum_{q=q_{\min}}^{q_{\max}} \left(\frac{y(q) - \mu(q)}{\sigma(q)}\right)^2 \tag{7}$$

When applying the Anscombe Transform to normalize Poisson distributed data in good approximation, the asymptotic bias of $-\frac{1}{4}$ is added to the model function and variance unity is assumed for the transformed data:

$$\begin{aligned} y(q) &= 2\sqrt{I_{\text{det}}(q) + \frac{3}{8}}, \\ \mu(q) &= 2\sqrt{I_{\text{fit}}(q) + \text{bias} + \frac{3}{8}} \approx 2\sqrt{I_{\text{fit}}(q) + \frac{1}{8}}, \text{ and} \\ \sigma(q) &\approx 1 \end{aligned} \tag{8}$$

When plugging the transformed quantities from (8) into (7), the least-squares minimization objective as previously given in Equation (2) is obtained:

$$\chi^2 = \sum_{q=q_{\min}}^{q_{\max}} \left(2\sqrt{I_{\text{det}}(q) + \frac{3}{8}} - 2\sqrt{I_{\text{fit}}(q) + \frac{1}{8}}\right)^2 \tag{9}$$

For comparison, the Poisson negative Log-Likelihood to minimize is given by (Lass *et al.*, 2021):

$$-\log \mathcal{L} = \sum_{q=q_{\min}}^{q_{\max}} I_{\text{fit}}(q) - I_{\text{det}}(q) \cdot \ln[I_{\text{fit}}(q)] + \ln[I_{\text{det}}(q)!] \tag{10}$$

The term $\ln[I_{\text{det}}(q)!]$ is constant and can be omitted during minimization.

6.3 Bias due to transformation and estimated variances

The weighted least-squares optimization goal for data with mean y_n and variance σ_n^2 with a model function μ_n is defined as $\chi^2 = \sum_n \left(\frac{y_n - \mu_n}{\sigma_n}\right)^2$. (Nelson & Prescott, 2019; McCluskey *et al.*, 2023)

In a similar manner as Lass *et al.*, we determined the expected value for a weighted least-squares-fit μ for random Poisson distributed samples $X \sim Po(\lambda)$ (Lass *et al.*, 2021). The difference $\mu - \lambda$ is the bias which is displayed in Figure 7. The value of the weigthed fit will approach a value of $\mu = \frac{\sum_X^\infty (\frac{p_\lambda(X)}{\sigma^2(X)} X)}{\sum_X^\infty \frac{p_\lambda(X)}{\sigma^2(X)}}$ with the Poisson probability distribution $p_\lambda(X) = \frac{\lambda^X e^{-\lambda}}{X!}$ for the expected value λ. For transformations of the data, X and σ^2 are substituted with their transformed values and the μ is inversely transformed to obtain the expected fitting result.

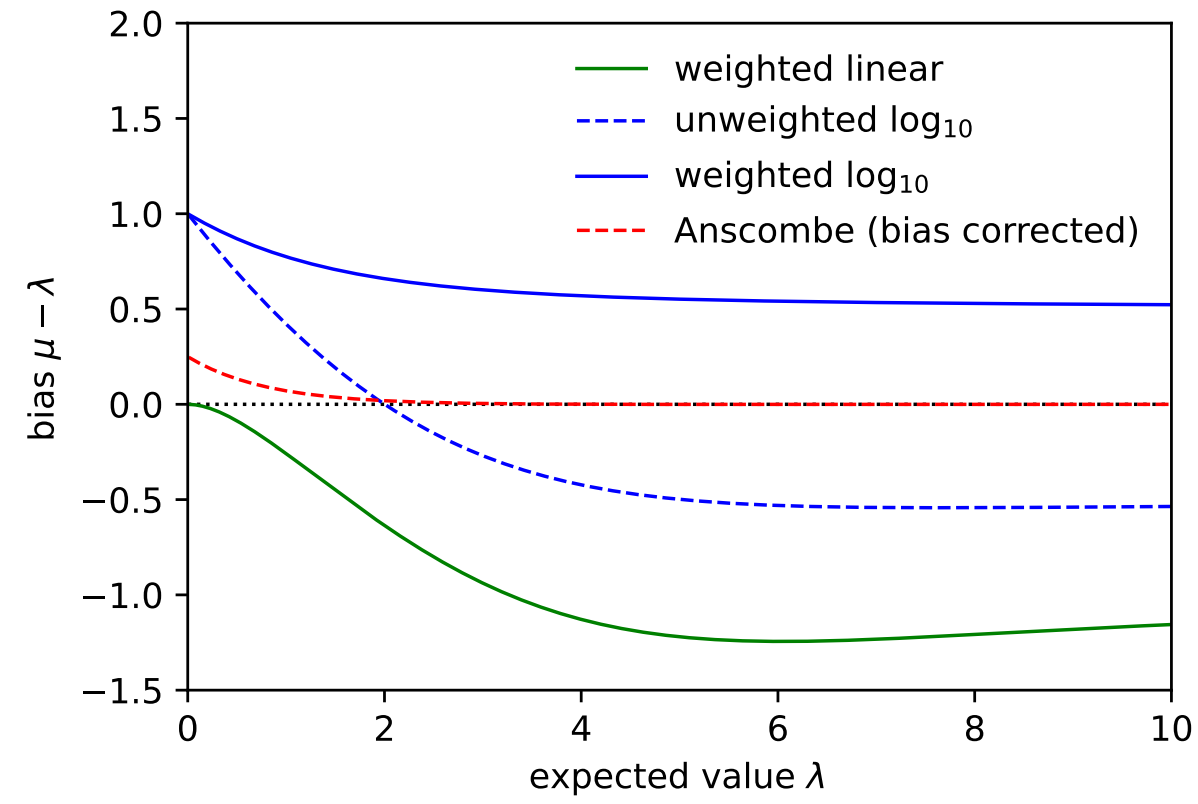

Figure 7: Illustration of the bias introduced by least-squares fitting a (transformed) Poisson distributed variable X with expected value λ. Probabilities for counts up to 1000 were evaluated.

Figure 7 indicates that the logarithmic transform introduces a positive bias close to 1 for low λ when counts of zero are discarded and approaches 0.5 or -0.5 for larger λ when weighted or unweighted, respectively. In the linear weighted case when estimating the variance $\sigma^2 = X$ and $\sigma^2 = 1$ for counts of zero, a negative bias close to -1 is observed for larger λ.

The shapes of transformed normal distributions for $\lambda = 3$ are displayed in Figure 8.

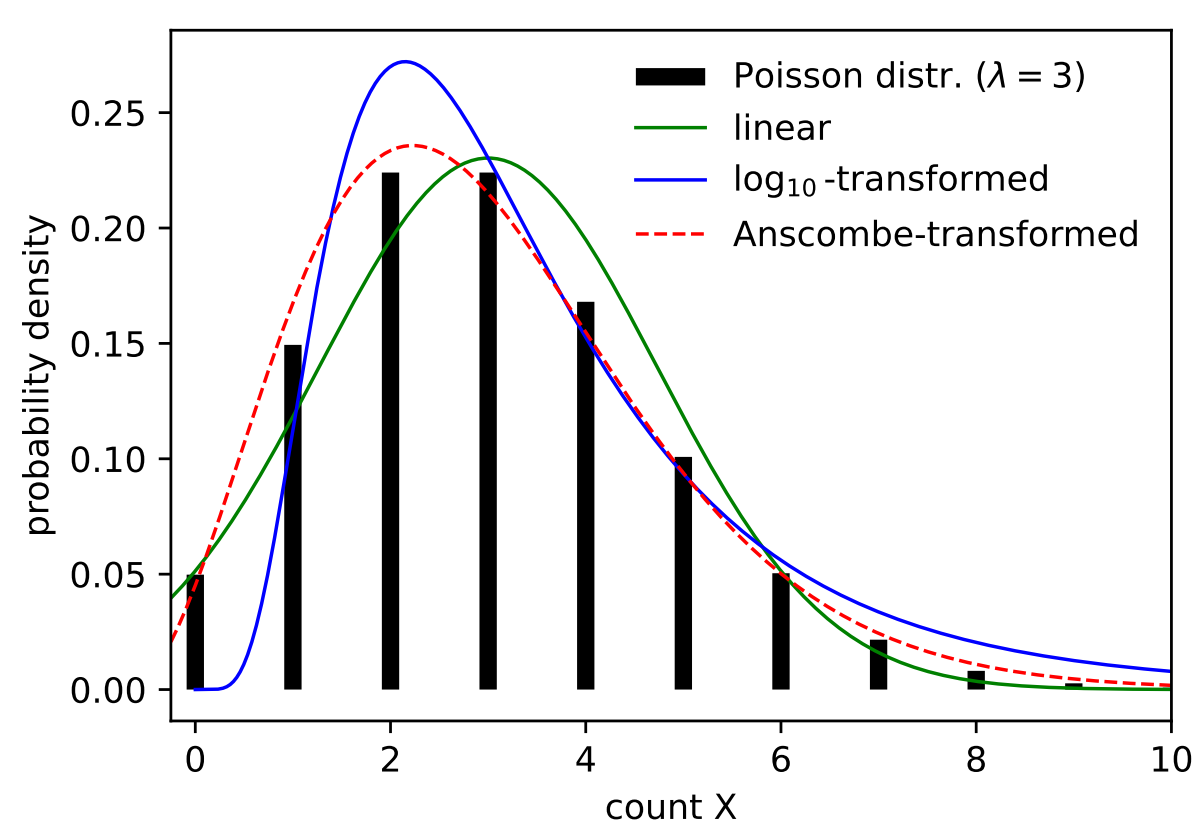

Figure 8: Approximation of the Poisson distribution for $\lambda = 3$ with different normal distributions $\mathcal{N}(\mu, \sigma)$ on a scale Y: Linear scale ($Y = X$, $\mu = \sigma^2 = \lambda$), logarithmic scale ($Y = \log_{10} X$, $\mu = \log_{10} \lambda$, $\sigma = \frac{\sqrt{\lambda}}{\lambda \ln 10}$) and the Anscombe transformed scale ($Y = 2\sqrt{X + 3/8}$, $\mu = 2\sqrt{\lambda + 1/8}$, $\sigma = 1$), respectively. Probability densities were scaled for display on the linear scale, with a factor of $\frac{\mathrm{d}Y}{\mathrm{d}X} = \frac{1}{X \ln 10}$ for the log-transformed scale and a factor of $\frac{\mathrm{d}Y}{\mathrm{d}X} = \frac{1}{\sqrt{X+3/8}}$ for the Anscombe-transformed scale.

6.4 Fitting Results

The fitting results of selected scans used in the creation of figures for this publication are given in Table 1 below.

Corner plots of MCMC sampling runs are displayed in figure 9.

Table 1: Fitting results (median ± estimated sigma) of selected scans after MCMC sampling of an initial least-squares fit with **refnx**. If not otherwise specified, the normalized data was log-transformed prior to fitting to a maximum of $q_z = 0.35\,\text{Å}^{-1}$.

	Scale	Au thickness [Å]	Au SLD [10^{-6} Å^{-2}]	Au roughness [Å]	Cr thickness [Å]	Cr roughness [Å]	Si roughness [Å]
Initial value	1	100	100	2	80	2	1
Parameter boundaries	[0.5, 1.5]	[100, 400]	[50, 200]	[0.1, 10]	[60, 100]	[0.1, 10]	[0.1, 15]
236 s, conventional XRR (1 s/point)	1.0172 ± 0.0003	304.6722 ± 0.0013	126.441 ± 0.011	5.3154 ± 0.0005	82.374 ± 0.004	2.5829 ± 0.0009	5.992 ± 0.003
9.95 ms, triangle	1.072 ± 0.006	304.04 ± 0.04	126.5 ± 0.2	5.61 ± 0.02	83.1 ± 0.2	2.78 ± 0.04	6.97 ± 0.12
2.45 ms, triangle	1.050 ± 0.012	303.82 ± 0.08	126.3 ± 0.5	5.62 ± 0.04	82.7 ± 0.4	2.90 ± 0.07	7.1 ± 0.2
950 µs, triangle	1.04 ± 0.02	304.38 ± 0.11	128.1 ± 0.8	5.06 ± 0.04	80.9 ± 0.5	3.80 ± 0.09	7.0 ± 0.3
950 µs	1.02 ± 0.02	304.28 ± 0.14	125.2 ± 0.9	5.18 ± 0.05	82.6 ± 0.7	4.13 ± 0.11	7.1 ± 0.5
450 µs	1.04 ± 0.03	304.4 ± 0.2	126.2 ± 1.2	4.93 ± 0.07	83 ± 5	4.2 ± 0.2	7 ± 3
213 µs	1.07 ± 0.05	304.2 ± 0.4	128 ± 2	4.81 ± 0.10	81 ± 4	5.1 ± 0.3	10 5± 3
213 µs (weighted linear)	0.87 ± 0.04	303.8 ± 0.4	124 ± 2	6.2 ± 0.2	78 ± 4	3.6 ± 0.2	9 ± 2
213 µs (Anscombe transf.)	1.02 ± 0.04	304.0 ± 0.3	127 ± 2	5.8 ± 0.2	81.3 ± 1.3	3.5 ± 0.3	6.8 ± 1.1
213 µs (Poisson log-likelih.)	1.01 ± 0.04	303.9 ± 0.3	126 ± 2	5.76 ± 0.14	81 ± 2	3.4 ± 0.2	7 ± 2

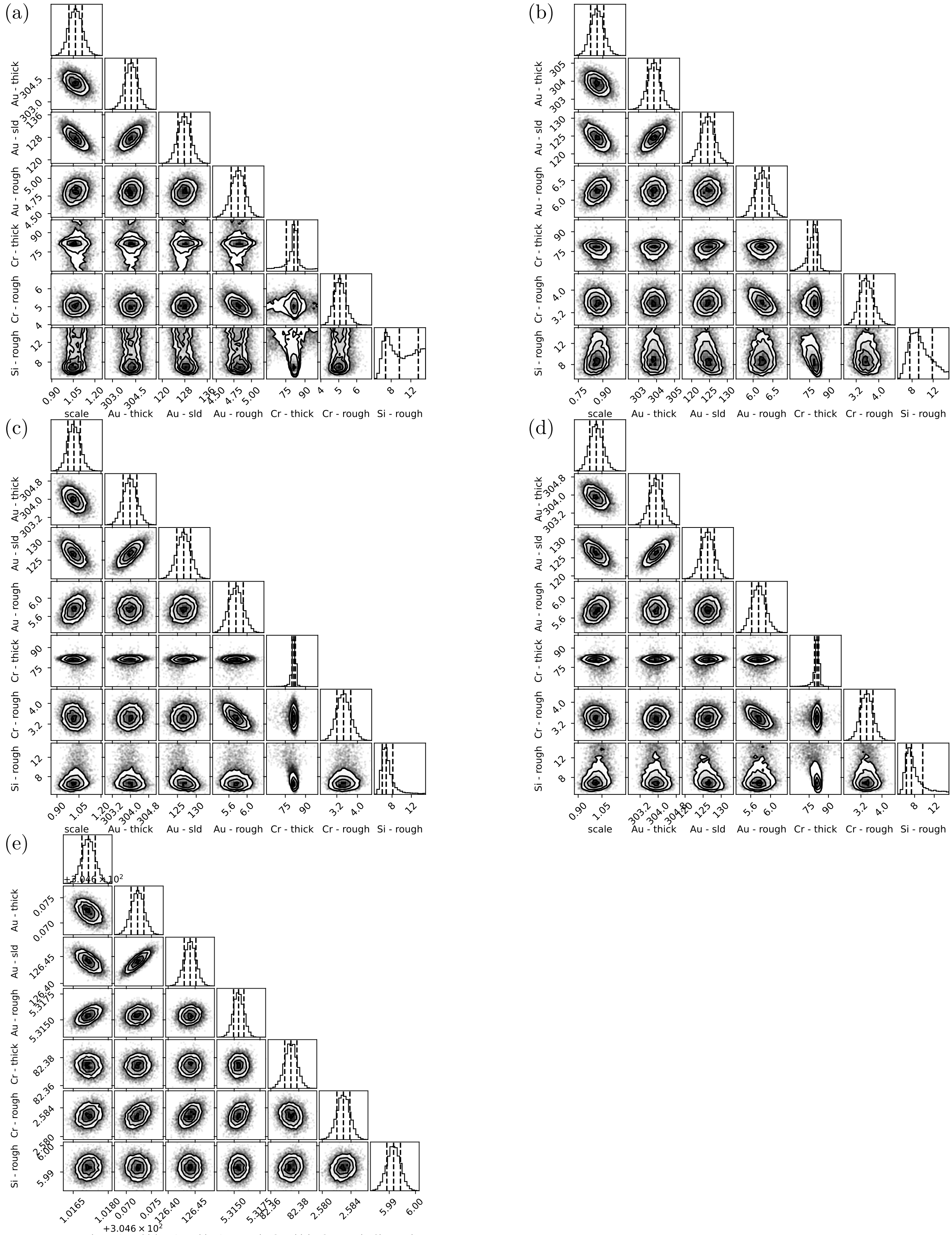

Figure 9: Corner Plots for MCMC sampling results of (a) $\log_{10}$-transformed, (b) untransformed, (c) Anscombe transformed, (d) Poisson Log-Likelihood fitted 213 µs scan and (e) 236 s conventional XRR scan
